# *De Novo* Fibrillary Glomerulonephritis (FGN) in a Renal Transplant with Chronic Hepatitis C

**DOI:** 10.1155/2013/978481

**Published:** 2013-06-13

**Authors:** Edward J. Filippone, Christine Chmielewski, Rakesh Gulati, Eric Newman, John L. Farber

**Affiliations:** ^1^Department of Medicine, Thomas Jefferson University, 2228 South Broad Street, Philadelphia, PA 19145, USA; ^2^Division of Nephrology, Thomas Jefferson University, Philadelphia, PA 19107, USA; ^3^Department of Pathology, Thomas Jefferson University, Philadelphia, PA 19107, USA

## Abstract

Chronic hepatitis C viremia (HepC) has been associated with numerous renal manifestations both in native kidneys and in the setting of renal transplantation. Glomerulonephritis (GN) of the renal allograft in the setting of HepC most commonly manifests as type 1 membranoproliferative GN (MPGN), either representing recurrence of the original disease or arising *de novo*. Other GNs were reported after transplantation in the patient with HepC including membranous nephropathy and thrombotic microangiopathy, as well as an enhanced susceptibility to transplant glomerulopathy. We describe the first case of *de novo* fibrillary GN in a renal transplant patient with HepC where the primary renal disease was biopsy proven type 1 MPGN. We discuss this relationship in detail.

## 1. Introduction

Chronic hepatitis C (HepC) affects 170 million persons worldwide and is a leading cause of cirrhosis and hepatocellular carcinoma. Renal disease is a prominent extrahepatic complication of  HepC, typically manifesting as membranoproliferative glomerulonephritis (MPGN) in the setting of cryoglobulinemia (cryo) [1]. In addition, HepC remains a major concern after renal transplantation, affecting both patient and graft survival [2]. A strong association is documented with development of posttransplant diabetes mellitus, *de novo* glomerulonephritis, and possibly transplant glomerulopathy. Here we report a patient with HepC and biopsy proven MPGN, who progressed to end-stage, and after 2 years on dialysis received a deceased donor renal transplant. Eight years later, in the setting of proteinuria and declining renal function, transplant biopsy revealed *de novo *FGN. 

## 2. Case Report

**Figure 1:**
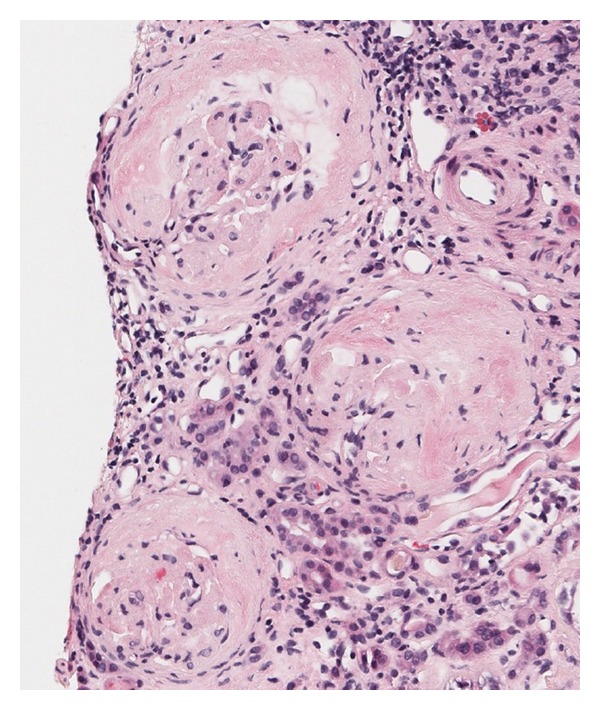
Photomicrograph of the kidney biopsy showing 3 glomeruli with prominent and advanced accumulation of amorphous, eosinophilic material (H & E, 40x).

**Figure 2:**
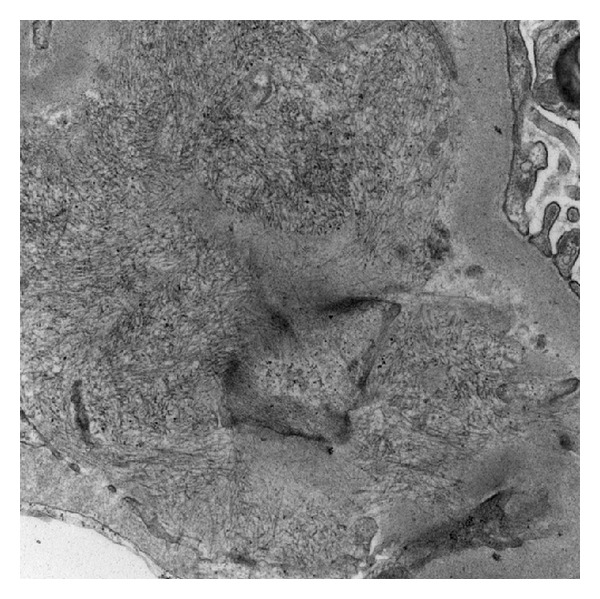
Electron micrograph showing an accumulation of randomly oriented, 18 nm in diameter fibrils in the subendothelial space between the lamina densa (upper right) and the endothelial cell (bottom left). Magnification: 20,000x.

A 56-year-old Caucasian male with HepC presented in 1994 with an elevated serum creatinine and nephrotic-range proteinuria. He had a history of hypertension, nephrolithiasis, and diverticulitss. Renal biopsy revealed type 1 MPGN. Symptomatic cryoglobulinemic vasculitis (cryoV) developed, which was treated in 1995 with plasmapheresis, intravenous cyclophosphamide, and steroids. Renal function progressively deteriorated necessitating dialysis in 1996. Treatment with interferon was curtailed, owing to severe psychiatric symptoms, and the patient remained viremic and on dialysis. A deceased donor renal transplant was performed in April 2003. An early Banff 1A rejection was treated with a course of IV methylprednisolone with resolution. Immunosuppressive therapy included cyclosporine and sirolimus. A biopsy in June 2003 showed no signs of rejection. In July 2003 cryoV reappeared with asthenia, severe arthralgias, purpura, and abdominal pain. The patient was viremic and hypocomplementemic with a cryocrit from 2 to 5%. Treatment included plasmapheresis and a 4-week course of rituximab, which produced symptomatic improvement. The serum creatinine ranged from 1.8 to 2.5 mg/dL from 2003 until 2010, when it increased to 4.5 mg/dL. A biopsy in June 2010 showed moderate acute tubular damage and mild chronic calcineurin inhibitor toxicity. Electron microscopy revealed a mild glomerulopathy consistent with calcineurin inhibitor toxicity. No evidence of recurrent MPGN was present. Dialysis was required for several weeks, but renal function spontaneously improved, and creatinine stabilized around 2.5 mg/dL. Over the course of the next year, creatinine slowly increased and proteinuria became nephrotic range. The protein/creatinine ratio was 4991 mg/g; SPEP showed decreased albumin and UPEP a pattern consistent with glomerular proteinuria. Both serum and urine immunofixation were negative. ANA was negative. HCV RNA PCR revealed 17,369,084 IU/mL. Serum cryo was negative, although they had been intermittently positive at 1-2%. C3 was normal and C4 was undetectable. A biopsy in December 2011 showed prominent and advanced accumulation of amorphous, eosinophilic material in glomeruli, interstitial fibrosis, tubular atrophy, and vascular intimal sclerosis (Figure 1). Electron microscopy showed 10–15 nm fibrils in the mesangium and glomerular capillary walls (Figure 2). A Congo red stain was negative. Renal function progressively deteriorated and dialysis was initiated in March 2012.

Our patient is the first reported case of FGN in a renal transplant developing in the setting of HepC. The morphologic features of the final transplant biopsy are clearly different from those of the native kidney (type I MPGN) and, thus, indicate a *de novo* process.

## 3. Discussion

HepC has been associated with various renal manifestations in both native and transplant kidneys. HepC is the cause of the large majority of cases of essential mixed cryo (EMC), whether due to type II (monoclonal IgM against polyclonal IgG) or type III (polyclonal IgM against polyclonal IgG) cryo [3]. This B-cell proliferative disorder is frequently complicated by glomerulonephritis (GN). About 75% of cases of GN complicating EMC are found with type II cryo and about 25% with type III [4]. Histologically, the lesions are a type 1 MPGN. Features that suggest an EMC etiology include prominent monocytic inflammation, large hyaline thrombi, and organization of deposits by electron microscopy (EM). Our patient's initial clinical presentation and native kidney biopsy clearly fit this description of cryo GN.

In a seminal report Johnson et al. found MPGN in all 8 HepC positive patients referred for evaluation of proteinuria [5]. Only 5 of the 8 had detectable cryo, although all 8 had low serum complement and positive RF. Organized deposits consistent with cryo were found in 3 of 4 examined specimens. In a follow-up study involving 34 HepC patients with proteinuria, 31 had MPGN and 3 had an acute proliferative and exudative GN [6]. Only 20 of 34 had cryo detectable on presentation. Over time, however, 9 more became positive (29 of 34 total). Twenty of the 29 positive patients had symptoms of EMC. Others have confirmed the association of HepC with MPGN [7, 8]. Most but not all of the reported cases had detectable cryo. In a series of 105 biopsies of native kidneys, Cosio at al. did not find an increased incidence of HepC in noncryo MPGN and concluded that the relationship between HepC and MPGN only holds in the presence of EMC [8]. 

Other glomerulopathies have been associated with HepC. Membranous nephropathy (MN) is documented in case reports [9]. Unlike MPGN, cryo and RFs are usually not found, and serum complement is usually normal. FGN or immunotactoid GN has been associated with HepC in several case reports [10–12]. In a series of 6 patients (4 with FGN and 2 with ITGN) reported in 1998, all were negative for cryo on presentation [13]. One patient, however, did develop them over time. This is somewhat analogous to our patient who had intermittent positivity. In a follow-up report in 2003, of 67 patients (61 with FGN and 6 with ITGN), HepC was found in 17% [14]. By contrast, however, in another series of 66 patients with FGN, only 3% had HepC [15]. In the report from Cosio et al. [8], HepC was significantly more frequent in patients with focal segmental glomerulosclerosis (FSGS) than in controls, but this was only true for intravenous drug users. In a series of 303 Egyptian patients presenting with GN, HepC was found in 38% [16]. This contrasts with a rate of 16% in the general Egyptian population. Of the 50 HepC patients from this series, 18 had type I MPGN, 9 type II MPGN, 12 FSGS, 2 MN, and 9 mesangioproliferative GN. Cryo were found in 27 of these 50 patients.

In order to establish the causality of HCV in these various disorders, numerous groups have sought evidence in renal tissue of HCV RNA by polymerase chain reaction (RCR) or *in situ* hybridization (ISH), as well as detection of viral proteins by indirect immunofluorescence (IIF) or immunohistochemistry (IH). In the Egyptian series noted above, IH for viral proteins was negative in all patients studied [16]. Electron microscopy detected particles consistent with HCV in 50% of cases, and PCR detected RNA in 4 of 21 cryo patients and 5 of 21 noncryo ones. Sansonno et al. studied 12 HepC patients with MPGN and type II cryo [17]. After electroelution, HCV-related proteins were detected in 8 of 12 patients by IH; all 8 HCV negative patients with MPGN had no positive reactions. Using laser capture microdissection to extract glomeruli from 20 HepC patients with various histologic types, the same group found HCV detectable RNA in 65% of glomeruli [18]. Positivity ranged from 33% of glomeruli in patients with IgA nephropathy to 83% of glomeruli in those with MPGN; HCV core protein was detected in an equal number of glomeruli as was RNA within each histologic type. Okada et al. detected HCV core protein by IIF in 2 patients with MN (both negative for RF and cryo) [19]. Cao et al. used IH to detect the NS3 protein in 21 HCV antibody positive patients with various types of GN [20]. This antigen was detected in 6 of the 21 (3 MPGN, 1 IgAN, 1 MN, and 1 amyloid); only 4 of the 6 were serum RNA positive. Immunoelectron microscopy showed that the antigen was localized mainly in electron dense deposits and in amyloid fibrils. Finally, in a study of 9 HepC MN patients, viral RNA (both genomic and replicative) and protein were detected in the perinuclear area of tubular epithelial cells by ISH and IH and were associated with greater interstitial inflammation/fibrosis compared to HCV negative MN patients [21]. It remains unclear if HCV has a causal role in these various glomerulopathies, with the exception of type 1 MPGN in the setting of EMC.

The incidence of HCV in patients on dialysis varies between 10 and 50% [22]. The same rate applies to patients who have received a renal transplant. Numerous studies have shown reduced patient survival after renal transplantation in case of HepC. This increased mortality may be due to posttransplant diabetes (strongly linked to HepC), cardiovascular disease, liver disease, and/or infection [2, 22]. Graft survival is also shortened by HepC. It still remains beneficial to receive a transplant as opposed to remaining on dialysis.

Proteinuria and the development of *de novo* glomerulopathy have been ascribed to HepC in renal transplantation. In a 1998 study of 322 consecutive transplants, Hestin et al. showed by multivariable analysis that HepC conferred a significant relative risk of 5.36 on the development of persistent proteinuria (>1 g/day for 3 months) [23]. Roth et al. studied 8 patients with proteinuria exceeding 1 g/day from a cohort of 98 HepC renal transplants [24]. Three had what was previously termed as chronic allograft nephropathy (CAN), but the other 5 had *de novo* development of type 1 MPGN. All 5 had undetectable cryo, but 2 did have low complement levels and positive RFs, and 2 patients also had organized deposits on EM. Cruzado et al. found 9 patients with >1.5 g/day proteinuria from a series of 94 HepC renal transplants [25]. One patient had MN, 2 CAN, and the remaining 6 *de novo* type 1 MPGN. All 6 did have detectable cryo, but none were symptomatic. IgM-RFs were detectable in the cryoglobulins, but 5 of the 6 had negative serum RF. HCV RNA was concentrated in the cryoglobulins, with enrichment of up to 18,000%. The same group evaluated 96 transplant biopsies performed at least 3 months after transplantation that did not show acute rejection [26]. Forty-four of the 96 (46%) were from HepC patients, and 63% of these showed *de novo *glomerulopathy (20 MPGN and 8 MN). This compares to 14% in those negative for HCV (3 MPGN, 4 MN). Similarly, Özdemir et al. studied 165 renal transplants of whom 44 were HCV antibody positive [27]. Of the 24 RNA+ patients, 15 developed *de novo* glomerulopathy (11 MPGN, 4 MN); only 8 of the 121 HCV negative patients did so. No case had detectable cryo. However, in a series of 2000 renal transplants (400 HepC), 15 cases of MN were detected, 10 of which were clearly *de novo*; no MPGN was apparently detected in this large series [28].

Other glomerular manifestations of HCV have been reported in renal transplants. Cosio et al. noted HCV in 29% of 41 patients with acute transplant glomerulitis and 33% of 27 patients with transplant glomerulopathy (TG) compared to 1.8% of 105 transplant patients with neither one [8]. Baid et al. studied 18 HepC transplants out of 339 total transplants [29]. Five of these 18 developed a thrombotic microangiopathy (TMA) a mean of 14 days after transplantation. All 5 had detectable anticardiolipin antibodies, as opposed to 1 of the 13 without TMA and 0 of 7 TMA patients without HCV. In a series of 209 indication biopsies for chronic renal allograft dysfunction from the same institution, 25 cases of TG were detected [30]. HepC was detected in 36% of these compared to 7% in a control group with calcineurin inhibitor nephrotoxicity and 3.6% in their overall transplant population. A TMA was detectable in 32% of the TG patients as well, and there was significant overlap between HCV and TMA in the setting of TG.

Glomerular organized deposits detected by EM as in our patient may be nonspecific findings or may be diagnostic of various conditions [31, 32]. Amyloid fibrils of any etiology are randomly oriented, nonbranching, and usually measure about 10 nm in diameter. Congo red positivity with green birefringence under polarized light is diagnostic, as is staining with thioflavin T. Cryoglobuins are frequently organized, as noted above, and may take various shapes depending on the type involved; interestingly, EM examination of cryoglobulins obtained from serum may mimic the morphology of those deposited in tissues [33]. The immune complexes of systemic lupus may occasionally be organized, appearing to resemble fingerprints [32]. 

The lesion termed as FGN, found in about 1% of renal biopsies, is characterized by the EM findings of randomly oriented, nonbranching fibrils about twice the diameter of amyloid (about 20 nm) [14, 15, 31, 32]. They are usually found in the glomerular basement membrane or paramesangial area. Tubular deposits are distinctly unusual, and except for isolated case reports they are not found outside the kidney. They are Congo red and thioflavin T negative but regularly stain for immunoglobulins, usually IgG (especially subclass IgG4), and complement by immunofluorescence (IF). Various light microscopic features may be obtained, including MPGN, mesangial proliferative GN, MN, and diffuse sclerosis. Patients present with hematuria, proteinuria, and renal impairment, and about half progress to ESRD in 2–4 years. It may recur following renal transplantation in about 50% of cases, but it tends to progress at a much slower rate in the transplant [34]. The largest series reported to date found an underlying  paraproteinemia (PP) in 15–17% of such cases [15]. Some authors feel that detection of a PP excludes a diagnosis of idiopathic FGN, suggesting that the fibrils are a manifestation of the underlying plasma or B-cell disorder [35]. This remains an area on contention. 

Related to FGN, but occurring about one-tenth as frequently, is ITG. This has a similar clinical presentation, light microscopic findings, and IF (except more commonly monoclonal) but differs by EM. Here, the fibrils are larger (usually, but not always, >30 nm), hollow (when viewed under 30,000x power or less), and/or organized (at least focally) into bundles. Patients with such deposits are more likely than those with the fibrils of FGN to have an underlying PP and/or B-cell neoplasm, occurring in over 50% of cases. This has led to the argument that FGN and ITG should be viewed as discreet entities. Others feel, however, that after excluding PPs, there would be no difference between them other than the diameter of the fibrils [34]. Neither FGN nor ITG frequently occurs *de novo* in renal allografts. Besides our case, we know of one case of *de novo* ITG associated with CMV infection [36] and another in a patient with systemic lupus [37]. One case of FGN from a large series was presumed to be *de novo*, although there was no native kidney biopsy [14].

As noted above, hepatitis C has been associated with FGN and is clearly associated with cryo. Since cryoglobulins are frequently organized, the fibrils detected in our patient, and other cases with HCV, may simply be cryoglobulin deposits. Indeed, it has been hypothesized that FGN may represent a forme fruste of cryo [13, 34]. Although our patient's initial biopsy had the more characteristic lesion of type 1 MPGN, the final transplant biopsy clearly did not. It is possible that after transplantation, due to immunosuppressive medications and/or effects of the allograft itself, the antigen/antibody ratio or other features of the immune complexes have been altered such that fibril formation was fostered. Perhaps in some patients with HepC this may occur from the outset, resulting in typical FGN in the native kidney. Finally, HCV-related EMC is definitively a B-cell proliferative disorder [38]. Considering the known association of such neoplastic disorders and PP with FGN and/or ITG [39], it may be that through the development of a neoplasm that HepC is linked to fibrillogenesis, independent of its ability to induce cryoglobulin formation. 

In conclusion, we present the first documented case of FGN developing *de novo* in a renal transplant patient chronically infected with HCV.
